# What we know when we act

**DOI:** 10.1007/s11098-023-01997-5

**Published:** 2023-06-24

**Authors:** Timothy Kearl

**Affiliations:** COGITO Epistemology Research Centre, University of Glasgow, 67-69 Oakfield Avenue, Glasgow, G3 8LP UK

**Keywords:** Inadvertent virtue, Know-how, Intentional action, Practical knowledge, Ability

## Abstract

Two traditions in action theory offer different accounts of what distinguishes intentional action from mere behavior. According to the causalist tradition, intentional action has certain distinguished causal antecedents, and according to the Anscombian tradition, intentional action has certain distinguished epistemological features. I offer a way to reconcile these ostensibly conflicting accounts of intentional action by way of appealing to “ability-constituting knowledge”. After explaining what such knowledge is, and in particular its relationship to inadvertent virtue and knowledge-how, I suggest that, among other things, appealing to ability-constituting knowledge can help us flesh out what it is for an agent’s reasons to non-deviantly cause and sustain her purposive behavior.

## Introduction

Two traditions in action theory offer different accounts of what distinguishes intentional action from mere behavior.

**The causalist tradition:** intentional action, as opposed to mere behavior, has certain distinguished *causal antecedents*. In particular, when an agent acts intentionally (to X, say), her beliefs, desires, and intentions cause her X-ing (in the right way).^1^

**The Anscombian tradition:** intentional action, as opposed to mere behavior, has certain distinguished *epistemological features*. In particular, when an agent X-s intentionally, she knows what she is doing as she does it.^2^

These traditions, as stated, may seem to place only necessary constraints on what it is to act intentionally, and, as such, one might wonder how they are in conflict at all. But that’s because a lot of important theoretical work is hidden in parenthetical clause “in the right way”. Causalists often speak as though *a non-deviant causal connection* between an agent’s behavior and her antecedent beliefs, desires, and intentions is both necessary and sufficient for that behavior to count as an intentional action. This, at any rate, is the kind of causalist that I have in mind throughout the essay.

According to (typically causalist) critics, Anscombians have a hard time explaining a range of very ordinary—even if not *paradigm—*cases of intentional actions, intentional actions performed in the absence of certain first-personal doxastic attitudes. For instance, when distracted drivers make it home from work, they do so intentionally, but it seems odd to describe them as “knowing what they are doing as they do it”, if for no other reason than they are distracted. And when athletes cannot explain why or how to perform complicated physical sequences, even as they do them intentionally, they plainly lack whatever knowledge underwrites an ability to explain what they are doing. For instance, competent cyclists often describe how to turn a bike one way but then go about turning another way. Their firm convictions expressed in *describing* how to turn do not count as knowledge, since those convictions are false.

Taken at face-value, each of these problematic cases involves an agent performing an intentional action in the absence of knowledge of what they are doing as they do it. And if Anscombian views have problems accommodating these cases, it seems reasonable to conclude *so much the worse for an epistemic constraint on intentional action*. One might suspect that, even if central or well-ordered cases of intentional action are ones in which the agent stands in a special epistemic relation to what they are doing as they do it, standing in such a relation is not particularly important to action theorists. In fact, Sarah Paul (2009) has argued that practical agents only have a kind of contingent, *inferential* knowledge of what they are doing as they do it. When an agent knows what she is doing as she does it, on Paul’s view, it is because the agent knows what she (previously) decided to do, and she can infer, perhaps by way of background beliefs concerning her own efficacy as a practical agent, that she is doing what she (previously) decided to do. Paul notes, rightly to my mind, that even if this sort of contingent, inferential knowledge of what one is doing is desirable and important for practical agents, it is not the mark of intentional action per se.

But cases of distraction or absent-mindedness, cases of inarticulability, and the like only threaten a fairly narrow band of Anscombian views.^3^ This fairly narrow band of views accept a highly intellectualized, discursive conception of the knowledge one has of what one is doing as one does it. Characteristically, discursive knowledge is the kind of knowledge one can *state* or *articulate*, one can *bring to mind*, one can *offer reasons in favor of*, and so on. This sort of knowledge is plainly valuable to practical agents, but we should all agree with causalist critics of Anscombianism that *it* is not the mark of intentional action per se.

What sort of knowledge is a better candidate? Below, I’ll argue for the following quasi-Anscombian position:

**Ability-constituting knowledge of action (“AKA”):** When an agent X-s intentionally, she manifests ability-constituting knowledge of action as she X-s.

After explaining what ability-constituting knowledge of action is, and in particular its relationship to knowledge-how, I focus on two chief virtues of accepting AKA. First, appealing to such knowledge is specially suited to distinguish between cases of “inadvertent”^4^ intentional action from merely unintentional ones. In this respect, my position fits into a unified account of inadvertent virtue across various normative domains, of which action theory is one.

Second, while the view I defend may naturally fit within the Anscombian tradition, it is consistent with views in the causalist tradition; in principle, there is nothing inconsistent about both accepting a causal theory of action and accepting AKA. In particular, one might think that an agent’s beliefs, desires, and intentions cannot cause her behavior *in the right way* (or “non-deviantly”) unless the way in question is itself a manifestation of ability-constituting knowledge of action. By appealing to an agent’s ability-constituting knowledge of action, causalists can explain how one’s antecedent and occurrent mental states causally sustain one’s subsequent behavior so as to count as “guiding” or “controlling” it.^5^ If one accepts AKA, one can offer an illuminating account of what this guidance consists in, and why it is intuitively absent in unintentional actions and mere behavior, in terms of the possession and manifestation of ability-constituting knowledge. Thus, to the extent that it is possible, my view reconciles causalism and Anscombianism.

So, what is ability-constituting knowledge of action, and how does it differ from other, more familiar kinds of knowledge?

## Ability-constituting knowledge and rational inference

In order to characterize ability-constituting knowledge of *action*, it helps to start with the nature and normative role of ability-constituting knowledge of *inference*.

**Ability-constituting knowledge of inference (“AKI”):** When an agent rationally infers proposition P from evidence E, she manifests ability-constituting knowledge of inference as she infers.

Ability-constituting knowledge of inference is not, for instance, knowledge *that* one has inferred; it is, instead, knowledge that “connects” evidence to hypotheses, doing so without itself serving as evidence.

Consider Carrol’s What the Tortoise said to Achilles. Therein, the tortoise asks Achilles what to make of an agent who knows that P, and that P implies Q, but who simply fails to see that Q. Achilles, naively, suggests that the agent may simply be missing a premise: that if (P and P implies Q), then Q, but he is then faced with the question of what to make of an agent who knows that P, that P implies Q, and that if (P and P implies Q), then Q, but who simply fails to see that Q. Achilles, absurdly now, suggests that the agent may simply be missing a premise. And so on. While this particular Carrollism has received much attention since its publication, often giving rise to divergent sophisticated analyses of what has gone wrong with Achilles’ suggestion, there is general consensus that Carroll has demonstrated something important about competence with rules of inference. What it is, in this case, to be competent with deduction is not to deduce by way of additional, perhaps suppressed premises.

Still, when a rational agent competently deduces Q from her evidence P and If P, then Q, she manifests her knowledge that If (P and if P, then Q), then Q*.* We should just deny that this knowledge is the sort of thing that serves as *further evidence* for judging that Q. Instead, this knowledge *guides an agent’s judgment* that Q from her evidence *P* and *if P, then Q*.^6^

Talk of “guidance” may prompt a variety of reactions, and I mean to use that term in a fairly deflationary sense. In general, it is not true that when one is guided by one’s knowledge (of the conditional If (P and if P, then Q), then Q, say), one can articulate the guiding knowledge, or “hold it before one’s mind”. Second and relatedly, “guidance” may operate merely implicitly. We should not think that the knowledge guiding an agent’s behavior is in principle accessible to the agent herself.^7^

One might wonder: what good is ability-constituting knowledge of inference? Why can’t frugal epistemologists get by with all and only *evidence-constituting* knowledge? For epistemologists, ability-constituting knowledge of inference plays at least one crucial normative role: that of determining whether an agent has *properly based her beliefs on her evidence*.

Imagine an agent, Ham, who believed both P and if P, then Q, and, in the course of considering whether Q, the agent is hit on the head with a hammer, and the precise force and angle of impact scrambles his brain so as to make him believe that Q, while retaining his earlier evidence. Despite “fitting” the evidence, there is something normatively deficient about Ham’s belief. Many authors would explain this deficiency as a failure of proper basing; the connection between Ham’s evidence and his judgment is just too *lucky* to credit the agent with any sort of epistemic achievement. Despite a kind of “fit” between evidence and inference, Ham does not infer Q *because of its connection to his evidence*. After all, being hit on the head at just the right angle so as to come to believe all and only the hypotheses supported by one’s evidence is patently not to manifest any sort of knowledge of the connection between evidence and hypothesis.

Sosa (2007; 2010) has long defended a sympathetic view. In his terms, we might say that Ham’s belief is irrational because, in failing to manifest Ham’s inferential abilities, that belief fails to be apt, or *accurate because adroit*. Being hit on the head with a hammer is not an adroit or skillful way to form accurate inferences on one’s evidence; after all, forming beliefs in this way often leads to error. Of course, one does not have to be a Sosa-style virtue epistemologist to accept these “ability constraints” on proper basing.^8^

In general, manifesting ability-constituting knowledge of inference rules out a form of *luck* in epistemology. This sort of luck diminishes the *agent’s contribution* to her behavior. If an agent with evidence E comes to believe hypothesis H but not by an exercise of her epistemic agency, her belief is too lucky, in this sense, to count as rational.^9^

So far, I’ve suggested that ability-constituting knowledge of inference connects evidence to hypotheses, but on pain of a Carrol-style regress, this knowledge cannot be further evidence (not on a natural construal of ‘evidence’, anyway). Moreover, that an agent manifests this ability-constituting knowledge of inference (that if E, then H) in moving from E to H explains why the judgment that H is attributable to the agent as a rational inference, not, for instance, as something that merely happens to her.

Below, I suggest that ability-constituting knowledge of inference and ability-constituting knowledge of action are species of a common genus: Ability-constituting knowledge. Species of this genus play a special role in various first-order normative theories: that of “linking” reasons to reasons-based performances. The knowledge that links reasons to reason-based performances is not itself a reason, on pain of a Carrol-style regress, and this knowledge plays an ineliminable role in explaining why certain inferences, despite “fitting” one’s evidence, are deviant.

## Ability-constituting knowledge and intelligent action

Why think that action theory needs the same conceptual resources as epistemology? More specifically, why think that action theory needs to “connect” reasons for action^10^ to action by way of manifesting some ability-constituting knowledge, as epistemology needs to connect premises to conclusion by way of ability-constituting knowledge?

The short answer is that we need to be able to explain why *some* cases of “inadvertently” successful performance are merely unintentional actions, while others are *bona fide* intentional actions. Here, “inadvertent” only serves to mark that the action in question is performed in the absence of a certain kind of knowledge, whatever knowledge would enable the acting agent to correctly judge or explain what they are doing as they do it. Consider, as an instance of cases of the first kind, the novice dart-thrower:

**Darts:** Al meets up with some friends at a local bar, *Che’s Lounge*. *Che’s* happens to have a lively darts competition one night each week, and it is slated to begin as Al finishes his second beer. Never having played a game of darts in his life, but brimming with confidence, Al signs up to play, fully intending to win. On his first turn he looks at the bullseye, makes some arm movements that, for all he knows, resemble the arm movements of genuine dart players, and sends the dart on a wing and a prayer. Lo and Behold! Al hits the bullseye. As it turns out, Al goes on to whiff every subsequent throw and loses badly.

In **Darts**, Al hits the bullseye, but he does not *intentionally* hit the bullseye. This should be an acceptable verdict for causalists and Anscombians alike. After all, Al has never played a game of darts before in his life, and it’s pretty obvious, after the game is over, that his first bullseye was beginner’s luck. We might say, of Al’s hitting the bullseye, that a stopped clock is right twice a day. (At any rate, this is the verdict often taken for granted in thinking about cases like **Darts**, and I will accept it for the sake of argument.^11^)

In claiming that Al’s hitting the bullseye is not an intentional action, I do not mean to deny that Al’s hitting the bullseye is an action *full stop*. For instance, there is a clear difference between Al’s hitting the bullseye as described in **Darts**, and Al’s hitting the bullseye as a result of being shoved in the direction of the dart board, or as a result of sneezing while noting the heft of a dart. If Al were to hit the bullseye as a result of these events, it would be odd to think that *Al* did anything to bring about the dart hitting the bullseye. In those sorts of cases, hitting the bullseye is just something that *happens to him*.

This simply brings out two ways in which an agent’s behavior may fail to count as intentional action: that behavior might count as action that is not intentional, or it might fail to count as action at all. Why think that, in **Darts**, Al’s hitting the bullseye counts as any kind of action, as opposed to something that merely happens to him? Short of answering this question with a complete action theory, one plausible thought is that Al’s hitting the bullseye is an action at all in virtue of other actions that Al performs intentionally: his throwing the dart at the board, perhaps his *trying* to hit the bullseye, and so on. In other words, the fact that Al does *something else* intentionally can explain why his hitting the bullseye counts as an action at all, albeit an unintentional one.^12^

Supposing that Al’s hitting the bullseye is an action, but one which falls short of intentional action, what ingredient is missing? For Anscombians, Al succeeds in hitting the bullseye in the absence of knowledge of what he is doing as he does it, and that accounts for our judgment that Al does not intentionally hit the bullseye. After all, he surely does not know that he is throwing his dart in a way that makes it likely to hit the bullseye, nor does he know anything about what he’s doing that would enable him to explain to others how to do it.

For causalists, Al’s success is somehow “causally deviant”; his mental states antecedent to throwing, which perhaps include his belief that the dart board is in front of him, his belief that dart players throw *thusly*, his desire to hit a bullseye, and his intention to hit a bullseye (by throwing *thusly*), constitute Al’s reasons for action. Those reasons for action somehow or another cause Al’s successfully hitting the bullseye, but however the causal story goes, it is not the right one to ground Al’s *intentionally* hitting the bullseye.

Now, compare Al’s case to Billy’s:

**Bike:** “Consider the following counterintuitive fact about cycling: to turn right, you typically start by steering left. All competent cyclists are in a position to deploy that information for the purposes of making turns. But few are able to deploy it for the purposes of explaining how to ride a bike. In fact, when giving verbal explanations, most cyclists are disposed to report exactly the opposite” (Elga and Rayo, 15-6). Billy, a competent cyclist, believes and reports that to turn right, you start by steering right. He then hops on a bike to demonstrate a right turn, and he starts by steering left. This inconsistency is lost on Billy.^13^

Clearly, Billy lacks knowledge of how to ride a bike that would enable him to correctly explain how to do so. He may have accumulated lots of misleading testimonial evidence that precludes him from being knowledgeable in this fairly specific way. But just as clearly, Billy nevertheless has whatever knowledge enables him to intentionally perform a right turn; he is, for instance, no more acting on *mere reflex* than any other competent cyclist in similar situations.

What could explain why, in **Bike**, the agent’s success is attributable to them as intentional action, but in **Darts** it is not? One answer suggests itself: not only does Billy intend to turn right, and not only does Billy’s turning right have a means-end structure, but, importantly, Billy manifests a special kind of knowledge in behaving as he does. To see why this last feature makes a difference, compare Billy to his brother, Willy. Willy, never having ridden a bike himself, has diligently documented and memorized all of Billy’s (mistaken) advice. Willy might hop on Billy’s bike and, having largely the same reasons as Billy did—certain beliefs concerning how one turns a bike, certain desires to avoid falling, certain intentions to turn—succeed in turning. But Willy’s case is importantly different than Billy’s. Insofar as Willy’s success is total beginner’s luck, it seems clear that Willy’s success in turning right is a merely unintentional action.

Why not say, though, that Willy’s success is intentional, like Billy’s? After all, Willy intends to turn right, and his turning right plausibly exhibits a means-end structure, insofar as Willy incorrectly believes that, in order to turn right, one starts by steering right. In short, the connection between what Willy intends to do and what Willy succeeds in doing is no more secure than the connection between what Al intends to do and what Al succeeds in doing. (Perhaps in light of these features we should grant that Willy intentionally *tries* to turn right.) What Willy lacks and Billy possesses is a special sort of knowledge: Billy knows, perhaps only implicitly, how to turn a bike, even if each has false explicit beliefs about what they’re doing.

Billy’s predicament in **Bike** bears some structural similarities to cases of inadvertent epistemic virtue in epistemology, and to cases of inadvertent moral worth in theories of moral responsibility. For instance, Brian Weatherson (2019) imagines that Aki is convinced of testimonial skepticism by powerful philosophical arguments. (Testimonial skepticism is the view that one cannot get reasons to believe propositions on the basis of testimony.) Aki’s friend, whom Aki has every reason to trust, tells her that *p*. Aki, despite her skeptical philosophical leanings, comes to believe that *p* on the basis of her friend’s testimony. According to Weatherson, that is what precisely what Aki should think, given the circumstances and her evidence. Her case is peculiar in that “she forms the right belief, for the right reasons, *while thinking these are bad reasons*” (170, emphasis added).

Nomy Arpaly (2002) has argued convincingly that theories of moral worth must explain what it is about Huckleberry Finn’s freeing Jim that distinguishes him from a merely “accidental good-doer”, and which elevates him above those “with liberal principles who are still viscerally prejudiced against people of different races” (10). Ultimately, her own explanation, developed in more detail in later work (Arpaly and Schroeder (2014)) is that Huck acts from intrinsic desires that are *de re* sensitive to the right-making features of action. Abstracting away from the details of her positive proposal, Huck’s case is one in which he does the right thing, for the right reasons, while thinking these are bad reasons.

If I’m correct, these similarities between cases of inadvertent epistemic virtue, cases of inadvertent moral worth, and **Bike** should not be surprising; rational judgments, morally worthy actions, and intentional actions are *reasons-based performances attributable to the agent*. Epistemic agents, morally responsible agents, and practical agents have, by virtue of the kind of things that they are, capacities to recognize and respond to reasons of certain sorts (epistemic, moral, and practical reasons, respectively).^14^ But it is not necessary, in any of these normative domains, for the agent responding aptly to reasons *to be able to explain what they are doing as they do it*, in order for us to attribute the reason-based performance to her. This rules out *one sort* of knowledge as necessary for the proper attribution of reason-based performances, but again, this is a fairly narrow and intellectualized conception of epistemic relation that agents stand in to their behavior.

## Ability-constituting knowledge as knowledge-how

So far, I’ve indicated that ability-constituting knowledge (of action or inference) is *not* the kind of knowledge one manifests by correctly explaining what one is doing as one does it. Instead, it may be merely implicit. Below, I’ll argue that it is best thought of as a form of knowledge-how.

Here is the general idea, hinted at in the last section: it is Billy’s manifesting his knowledge of how to ride a bike that renders his properly executed turn intentional, whereas Willy’s failure to manifest such knowledge (because he lacks it) renders his properly executed turn merely accidental. And recall Ham, who possesses P and if P, then Q as evidence, but only believes Q because he is hit on the head with a hammer; it is Ham’s failure to manifest his knowledge of how to perform simple, one-step deductions that renders his belief (that Q) irrational.

The idea that knowledge-how is intimately connected to intentional action is, of course, widely accepted.^15^ But the relationship between knowledge-how and rational inference tends to be neglected (or at least under-appreciated) in epistemology, and so the parallels between action theory and epistemology to which I am pointing go unnoticed.

To clarify my proposal, I want to address two, related concerns. First, Kieran Setiya (2008) has argued that we sometimes X intentionally without knowing how to X. Addressing Setiya’s position will offer an initial defense of **AKA**. Second, my account of ability-constituting knowledge *of inference* makes the relevant knowledge appear propositional, while my account of ability-constituting knowledge *of action* makes the relevant knowledge appear non-propositional; it may seem that there are really two things going on where I say there is only one. My aim is not, in the course of addressing these concerns, to offer a complete metaphysics of ability-constituting knowledge; rather, I hope to situate my proposal within a number of related debates.

### Setiya on action and inference

In “Practical Knowledge”, Setiya argues that we don’t have to know how to do everything that we do intentionally. In other words, one can *intentionally X* without *knowing how to X*. He says:

**Bomb:** “There are cases of intentional action that are not accompanied by knowledge how. For instance: I am trying to defuse a bomb, staring with confusion at an array of coloured wires. Which one to cut? In desperation, not having a clue what the wires do, whether they will trigger the bomb or not, I disconnect the red wire—and the timer stops. Even though I did not know how to defuse the bomb, and managed to do so through dumb luck, I count as having defused the bomb intentionally. That is certainly what I meant to do, despite my uncertainty.” (2008; 404)

In **Bomb**, the protagonist, call him “KS”, does not know how to defuse the bomb, in the sense that KS does not know which way of cutting the wires counts as a way of defusing the bomb. Setiya thinks that if KS goes about cutting wires, driven by something like his desire to avoid being blown up and his belief that cutting some wire or another might defuse the bomb, and thereby manages to succeed in defusing the bomb, there is some intuitive pressure to say that he intentionally defused it without knowing how.

Here is a simple way to make inroads on this rather dire case: either **Bomb** and **Darts** are relevantly similar, or they aren’t. If **Bomb** and **Darts** are relevantly similar, we should simply deny that Setiya has produced a *bona fide* case of intentional action. Even if, let’s suppose, KS’s bomb-defusing is merely unintentional, there is surely *something* that KS does intentionally (*trying to defuse the bomb*). But the connection between what KS does intentionally and his defusing the bomb is just too lucky for the latter to count as intentional too. If so, KS’s situation in **Bomb** resembles Al’s situation in **Darts**, and my view can explain why: KS and Al succeed merely unintentionally because each lacks knowledge of how to do that which they’re trying to do. That **Bomb** and **Darts** are relevantly similar is, I think, the most natural reaction; it is surely correct to think that KS’s defusing the bomb falls short of our paradigm of intentional action.

If, however, **Bomb** and **Darts** aren’t relevantly similar, it does not follow that **Bomb** is a case of intentional action, as Setiya suggests. One might treat KS’s defusing the bomb as a “middling action”, one that is neither intentional nor unintentional. Following Mele (1992) and Mele and Moser (1994), we could say that KS defuses the bomb “non-intentionally”. The category of non-intentionality applies, perhaps among other things, to “side-effect actions”, actions that, for all we know we might bring about without, strictly speaking, aiming to do so.^16^ Manifesting one’s knowledge of how to take certain relevant means (cutting a wire) to bring about a desired end (defusing the bomb), despite being largely ignorant of *which* of his available means will likely succeed in bringing about that desired end, may be enough to rise above the level of merely unintentional action without thereby counting as intentional. On my view, it matters quite a bit that KS’s knowledge of the means to defuse the bomb is incomplete but not entirely absent, while Al’s knowledge of the means to hit the bullseye is entirely absent.

We’ve just gone over two ways of denying Setiya’s claim that **Bomb** is a case of intentional action, and the plausibility of these denials, together with the natural explanation my account provides, relieves some dialectical pressure. But let’s consider, if only for the sake of argument, the possibility that **Bomb** is dissimilar to **Darts** because KS intentionally defuses the bomb. What, if not knowledge of how to defuse the bomb, could account for this verdict? For Setiya, it is knowledge of how to do something else:

“When I do something intentionally that I do not know how to do, I must at least know how to take some relevant means. In the present case, I know how to cut the red wire, and I think it might defuse the bomb, even though I can’t be sure.” (*ibid*)

Setiya’s suggestion is that X-ing intentionally requires knowing how to X, or else it requires knowing how to do something else (cutting the red wire, perhaps) that would count as way to X in the context. At this point, a friend of Setiya would need to say more about the relationship between knowing how to X, on the one hand, and the variety of more basic things one knows how to do that count as ways of X-ing in a context, on the other.

Without such an account on offer, one might reasonably wonder whether the right thing to say about **Bomb** is precisely what Setiya says about it (on the assumption that **Bomb** and **Darts** are dissimilar): that KS defuses the bomb intentionally *without knowing how to defuse the bomb*. Hear me out. Consider what happens in a significantly lower-stakes situation, as when you use the old Xerox machine in the office to make copies of a handout; you know that, in order to make copies on the old Xerox, you’ve got to hit either the red or green button, but you can’t remember which (the other button scans the document).^17^ You try the red one, say, and it works – copies abound! Here, for whatever it’s worth, I am not inclined to say that you’ve intentionally made copies *without knowing how to make copies*, even if we can all agree that your knowledge of how to make copies is incomplete. If this verdict is at all plausible in low-stakes situations, as when making copies, it is plausible in high-stakes situations, as when defusing bombs.^18^ The inclination to withhold the *attribution* of (more complex forms of) knowledge-how to KS, *if* one is so inclined, may simply reflect the stakes in his context, not of what KS in fact knows how to do.

Despite my disagreements with Setiya surrounding **Bomb**, one might suspect that there are only differences of detail or emphasis between our two positions.^19^ To the extent that our views bear a family resemblance, it is worth pausing to point out places at which they more sharply diverge, especially on the relationship between rational inference and knowledge-how.

According to Setiya, inference and action are two quite different beasts. In his “Epistemic Agency: Some Doubts”, he says:

“In the case of belief, believing that *p* and that the fact that *q* is evidence that *p* is sufficient for believing that *p* on the ground that *q*, and so believing that *p* because one believes that *q*; in the case of intentional action, doing Ф intentionally while believing that the fact that *p* is a reason to Ф is not sufficient for acting on the ground that *p* or because one believes that *p*… acting for a reason does not reduce to a mere conjunction of action and belief” (2013, 192).

On this conception, believing for a reason (unlike acting for a reason) is a matter of having the right conjunction of beliefs: all there is to my believing that *p* on the basis of *q*, Setiya thinks, is my believing *p* and believing that *q* supports *p*.^20^

But this sparse conception of believing for a reason (and, in particular, its implications for the nature of rational inference) is straightforwardly inconsistent with the possibility of inadvertent epistemic virtue discussed in the last section. Recall that cases of inadvertent epistemic virtue were ones in which an agent forms the right belief, for the right reasons, while thinking those were bad reasons.

Brian Weatherson’s suggestion is that, in cases of inadvertent epistemic virtue, an agent’s first-order evidence “evidentially screens-off” her judgments about her first-order evidence.^21^ That is not to deny that, in general, such judgments are evidentially relevant, nor is it to deny that “evidence of evidence is evidence”^22^; rather, it is to give voice to the thought that one’s first-order evidence has priority over one’s judgments about one’s evidence in determining what one ought to believe. Aki the akratic, convinced of testimonial skepticism, nevertheless finds herself with what is in fact strong first-order testimonial evidence to believe that P. Her strong first-order testimonial evidence that P evidentially screens-off her judgments about what her evidence supports. To borrow a phrase from Nomy Arpaly, Aki’s judgments *about* what her evidence supports play a rather superficial role in the drama of her rational inference.^23^

Of course, we don’t need to commit to Weatherson’s notion of evidential screening-off to appreciate the point that, in cases where an agent has misleading evidence *about* what her evidence is or what it supports, it is possible for an agent to believe that *p* on the basis of *q* while also believing that it’s not the case that *q* supports *p*. This is exactly Aki the akratic’s predicament, and this unfortunate conjunction of beliefs is a feature of cases of epistemic *akrasia* more generally.^24^

My own view is that Aki the akratic is inadvertently epistemically virtuous because, despite her misleading beliefs about what her first-order evidence is, her inference manifests ability-constituting knowledge, namely her knowledge of how to infer. Absent her manifesting such knowledge, there wouldn’t seem to be anything even *prima facie* rational about her inference, in which case she surely wouldn’t exhibit epistemic virtue;^25^ Aki would begin to look much more like Ham.^26^

### Species of a common genus

Having laid out my picture of ability-constituting knowledge in greater detail, we can address the second concern mentioned at the start of this section: it seems like, contrary to what I’ve suggested, ability-constituting knowledge of inference and ability-constituting knowledge of action are *not* species of the same genus. After all, in Sect. 2, I said that ability-constituting knowledge of inference, at least in cases of competent, single-premise deduction, was knowledge of the form *if (P and if P, then Q), then Q*. One might think that I am appealing to an especially important *proposition*, knowledge of which guides one’s inferences (at least in simple, single-premise deduction) so as to render them rational.

And in Sect. 3, I argued that certain cases of inadvertent intentional action are only distinguished from merely unintentional ones because the protagonist manifests ability-constituting knowledge of action, and I relied on certain classic examples of knowledge-how (like Billy riding a bike) to make this point. Many (but not all) accounts of knowledge-how treat it as essentially *dispositional* rather than propositional. Thus, one might think that I am appealing to an especially important *disposition*, the possession and manifestation of which guides one’s actions so as to render them intentional. A careful reader might wonder how these two ideas square with one another.

The first thing to emphasize in response to this sort of concern is that, even though ability-constituting knowledge of inference (for simple, single-premise deduction) can be represented by pointing to knowledge of an indicative conditional *If (P and If P, then Q), then Q*, this does not necessarily render such knowledge propositional.^27^ Again, it might simply be the most perspicuous way of representing that an agent *knows how to infer*. On this reading, the surface-level ‘propositionality’ of ability-constituting knowledge of inference is a red herring.

The second thing to emphasize is that I have been discussing the relationship between ability-constituting knowledge and knowledge-how, trying to set aside the relationship between knowledge-how and knowledge-that. As I see it, questions surrounding the relative metaphysical priority of knowledge-how and knowledge-that are orthogonal to whether there is a special form of knowledge-how, ability-constituting knowledge, necessarily manifested in rational inference and intentional action.

But the preceding discussion suggests that whatever knowledge-how is at bottom,^28^ extant accounts of knowledge-how are raised on a one-sided diet of cases: ones that are *action-* and *intention-centered*. If, however, we abstract away from the action- and intention-centeredness of the cases upon which accounts of knowledge-how are typically built, in part by bringing considerations of inference into the fold, then we might see ability-constituting knowledge as something like the kernel of knowledge-how, which, rather than having certain characteristic outputs (e.g., action as opposed to judgment), has certain characteristic normative functions: in particular, that of “linking” one’s reasons to one’s reasons-based performances in various first-order normative domains.^29^

Let’s briefly take stock. Short of offering a metaphysics of ability-constituting knowledge, I hope to have accomplished two lesser tasks. First, I hope to have characterized ability-constituting knowledge in terms of its normative role, that of connecting reasons to reason-based performances. Second, I hope to have shown that the thing playing this particular normative role is not suspicious. If ability-constituting knowledge (of both inference and action) is a special kind of knowledge-how, it could be understood in terms of propositional knowledge under a special mode of presentation, propositional knowledge indexed to certain contexts or aims, or something non-propositional, perhaps as a bundle of special dispositions (whichever turns out to be *the* correct view of the nature of knowledge-how and its relationship to knowledge-that, which, again, is a further question I have not attempted to address).^30^

## Summary

I have argued that one distinguishing mark of intentional action is that the agent knows what she is doing as she does it. The sort of knowledge in question is captured by AKA:

**Ability-constituting knowledge of action (“AKA”):** When an agent X-s intentionally, she manifests ability-constituting knowledge of action as she X-s.

Ability-constituting knowledge is not the kind of knowledge that serves as a reason to act or believe; rather, it guides these sorts of reason-based performances. For instance, competent cyclists often lack whatever knowledge enables them to correctly explain how to steer, while nevertheless knowing that one initiates a right turn by steering left; having this knowledge is part of what their competence consists in. That very knowledge may be merely implicit, inconsistent with one’s explicit judgments.

If my arguments are correct, I have identified a form of knowledge the manifestation of which is necessary for different kinds of reasons-based performances (in action and judgment) to count as “successes”. Moreover, I have shown that the manifestation of that knowledge is what explains why cases of inadvertent reasons-based performances are successes (in action and judgment), rather than things that merely *happen* to the agent. I have offered a way to think about ability-constituting knowledge in terms of knowledge-how, but this invites a number of questions about proprietary distinctions within extant accounts of knowledge-how, and I have, admittedly, only gestured at a complete answer.

Without the space to fully explore these ideas here, in closing I want to note two advantages of accepting AKA. The first should appeal to philosophers regardless of traditional commitments: AKA supplies principled reason to admit of an epistemic constraint on intentional action that side-steps familiar problems surrounding the nature of intention itself. The position developed here does not depend on any positive view about the nature of intentions, whether they are belief-like, desire-like, a hybrid belief-like and desire-like attitude, for instance. Nor does it depend on whether and to what extent the contents of one’s intentions must relate to (a particular description of) one’s actions in order to render those actions intentional (under that description). These are extremely important and interesting issues that any complete theory of action must address. But I hope to have shown that, whatever the final verdict on the nature and content of intentions per se, one must appeal to something like AKA to explain how intentions—however understood—could perform their characteristic *guiding* role.

The second advantage of accepting AKA should appeal, I think, primarily to causalists. Namely, AKA is consistent with views in the causalist tradition. This may come as a surprising result, given the way that causalist and Anscombian views are often pitted against one another. The apparent incompatibility of these traditions seems to be driven in no small part by an assumption that debates around an epistemic condition on intentional action are settled by considerations of the nature of intention per se. If my remarks above are correct, I have cast doubt on that assumption and opened up a section of previously unoccupied theoretical space, one that ostensibly inherits the benefits of both causal and Anscombian theories of action. Thus, to the extent that reconciliation possible, I have tried to reconcile causalist and Anscombian theories of action.

Why should this be appealing? Causal theories of action are *incomplete*, and just about everyone admits it; even if intentional actions necessarily have certain distinguished mental causal antecedents, causalists notoriously have problems with cases of *causal deviance*. What does its deviance consist in, or to put it differently, what would have to be added back into cases of deviance to turn them into cases of *bona fide* intentional action? On the simplest version of this causalist-*cum*-Anscombian view, an agent’s behavior counts as an intentional action only if her ability-constituting knowledge is itself a cause of that behavior. We might then say, for instance, that in **Bike**, Billy turns right intentionally because not only are his antecedent beliefs, desires, and intentions are a cause of his turning right, so too is Billy’s ability-constituting knowledge of how to turn right. On more complex versions of this kind of view, the ability-constituting knowledge is not itself a cause of Billy’s turning right, but it nevertheless grounds the relevant causal relations. We might then say that Billy’s ability-constituting knowledge grounds the fact that his intention to turn right causes his turning right. Whatever the details of the particular causal theory, AKA can help us say more about what it is to have the right kind of cause, rather than compete with such theories for an explanation of what it is to act intentionally at all.

## Footnotes

1 E.g., see Davidson (1971; 1978), Paul (2009), Mele (1992b; 2001), Bratman (1984; 1987).

2 E.g., see Anscombe (1957), Velleman (1989), Setiya (2009), Thompson (2011), Ford (2015), Schwenkler (2015), Campbell (2018), and Frost (2018). Anscombians accept (and causalists reject) that these epistemological features are that in virtue of which one’s behavior is intentional.

3 Notably, some authors have recently challenged Anscombe-inspired views by appealing to cases in which an agent intuitively acts intentionally while performing at a success rate that falls below a threshold for the reliability required for knowledge (see Carter & Shepherd (forthcoming-a; forthcoming-b)). I note these challenges to set them aside, as they would require taking a stand on the general relationship between knowledge and intentional action, on the one hand, and considerations of reliability or success rate, on the other.

4 I hope that the stipulated terminology is innocent enough; I mean to use “inadvertent” in the sense of “inadvertent virtue” or “inadvertent epistemic virtue” used by Arpaly (2002) and Weatherson (2019), according to which agents are inadvertently virtuous when they respond to the right reasons but do not know that they are so responding. It is natural enough, in ordinary contexts, to use “inadvertent” and “unintentional” as rough synonyms, and I break from that natural usage here.

5 Compare Schwenkler’s (2015) ambition to explain the connection between knowing one what is doing as one does it and the notion of “agential self-guidance.”.

6 I further characterize ability-constituting knowledge of inference in Sect. 4.

7 This, I take it, is no more controversial than the epistemological thesis that one’s mental life is not “luminous”. A mental state M is luminous (for agent A) just in case: when A is in M, A is in a position to know that she is in M. See, e.g., Williamson (2000).

8 See, for instance, Turri (2010), Markie (2013), Kearl (2022).

9 Of course, the predominant view about what it is for an agent to properly base her belief that P on her evidence E is that E must cause the belief that P (in the right way). Thus, a causal theory of basing faces the same deviance worries as a causal theory of action. (See, e.g., Turri (2010) for an extended defense and elaboration of this claim.) The most promising accounts of “non-deviant” basing consist not in rejecting a causal theory of basing, but in articulating constraints on which belief-formation mechanisms—understood causally—are rationality-grounding. A number of authors have suggested that these mechanisms must themselves be a kind of knowledge. See, e.g., Wedgwood (2013) and Silva (2017) for very different versions of this idea.

10 I mean to be ecumenical about how ‘reasons for action’ is best understood. Someone who is suspicious of the broadly Davidsonian framework I’m using could make do with talk of ‘intention’ instead. For instance, on a Bratman-style planning view of intention, intentions are *sui generis* psychological states with distinctive motivational profiles and connections to diachronic agency, and they would be ineliminable in the causal explanation of an agent’s behavior (alongside her reasons for action, explained in terms of her beliefs and desires). I hope that I can simply bracket this nuance for the sake of exposition.

11 See, e.g., O’Shaughnessy (1980), Harman (1986). Mele (1992a) officially registers agnosticism but offers similar cases of luck to undermine attributions of intentional action. Others, like Kenny (1975), suggest that the novice darts player lacks a pertinent kind of *ability*. Still others, perhaps Mele (1992b), might suggest that the novice lacks a pertinent kind of *control*. But it is plausible to think that ‘ability’ and ‘control’ are causal notions; for instance, it seems like the particular causal route by which the novice hits the board is not *control-grounding*, and it thereby falls short of counting as an intentional action.

12 One way to bring this out is to emphasize that Al’s hitting the bullseye counts as an action because there is some other *description of the event of his hitting the bullseye* under which Al brings it about intentionally.

13 This case bears some similarities to Bengson & Moffett’s *Salchow* (2007, 2011) and *Modified Salchow* (2011) examples, although I depart from their judgments about those examples and resulting position.

14 An anonymous referee points out that cases of inadvertent epistemic virtue and inadvertent moral worth involve agents who are mistaken about *what to believe* or *what to do, morally speaking*, whereas my cases of inadvertent intentional action seem to involve agents who are mistaken about *how to do* what they aim to do. This seems to put pressure on my claim that inadvertent successes (in rational judgment, morally worthy action, and intentional action) are all species of the same genus. The extent to which this is a disanalogy, I think, turns on the relationship between knowledge-how and knowledge-that, which I comment on briefly at the end of this section. For instance, on an intellectualist account of knowledge-how, Billy’s ability to ride a bike might be constituted by his knowing, of various ways *w*, *w’*, …, that *w* is a way to pedal, *w’* is a way to initiate turns, etc. Thus, Billy might be mistaken in his *de-dicto*, person-level judgments about which ways are ways to initiate turns, while still having knowledge about those matters in a *de-re* way. See, for instance, Pavese (2021). Supposing, however, that one rejects intellectualism, it still seems plausible that Huck manifests certain moral ability-constituting knowledge—for instance, to recognize the weight of Jim’s humanity and its relative importance to mores of the antebellum South. Huck manifests such knowledge despite being mistaken, at the level of his own deliberation, about how to weigh those considerations together. Ultimately, however, I am open to the idea that there are residual differences between the conditions that render intentional actions inadvertent, on the one hand, and the conditions that render morally worthy actions inadvertent, on the other, so long as there is a family resemblance between cases of these kinds.

15 For instance, see Setiya (2008; 2009; 2012), Löwenstein (2016), Stanley and Williamson (2001), Mele (1992a; 1992b), Mele & Moser (1994), Bengson & Moffett (2007), Hawley (2003), Hornsby (2012). Hornsby and Stanley & Williamson suggest that Ryle (1949) was in fact propounding a similar connection, albeit under the description of “intelligent” action.

16 Consider Harman’s (1976) example of the sniper who fires his gun trying to kill an enemy soldier, knowingly alerting the enemy to his presence. That he knowingly alerts the enemy to his presence in firing his gun does not entail that he intentionally alerts the enemy. But since his alerting the enemy is done knowingly and non-accidentally, there is surely pressure to resist labeling it a merely unintentional action. See Mele and Moser (1994, Sect. 3) for extended discussion.

17 Thanks to Carolina Sartorio for suggesting this example.

18 Granted, there are certain “pragmatic encroachment” views of knowledge-that according to which a difference in practical circumstances or stakes can constitute a difference in knowledge-that (Stanley (2005); Fantl & McGrath (2009)). That is already a highly contentious thesis regarding knowledge-that, and I cannot here assess the plausibility of an analogous claim about knowledge-how, but it seems fairly risky to anchor one’s arguments to this latter thesis.

19 In a footnote (2017, ch5, fn3), Setiya recognizes that one might preserve (a version of) the link I’m defending between intentional action and knowledge-how by appealing to *partial* knowledge-how, rather than to his disjunctive view, although he opts not to explore the former option. If he is sympathetic to appeals to partial knowledge, he may be at least sympathetic to treating KS’s bomb defusing as non-intentional, being the manifestation of *merely partial* or *incomplete* knowledge-how. He might also be sympathetic to the idea that manifesting incomplete knowledge-how can be enough for intentional action. At any rate, my aim is not to prove that Setiya is barking up the wrong tree; it is just to point out the theoretical space that exists between our two suggestions and certain considerations that might attract a neutral reader towards my position. To the extent that Setiya would be happy to countenance partial knowledge-how, my arguments may simply exhibit a novel route to a somewhat familiar position within action theory, and that’s fine by me. Still, our positions sharply diverge in epistemology, as I discuss below.

20 This view about inference bears a family resemblance to those that accept the “Principle of Inferential Justification”, due to Fumerton (1995).

21 He says: “facts about one’s own judgment are not evidentially relevant to what judgment one makes, provided one has access to the evidence behind that judgment. And that suggests that the judgment should really just be judged on how it tracks the evidence…” (2019, p. 198).

22 Comesaña and Tal (2015; 2017).

23 Arpaly suggests that one’s judgments about what one ought to do sometimes play a “rather superficial role in a drama in which a person is motivated by sinister [or virtuous, as the case may be] desires…” (2002, p. 106).

24 For more on epistemic *akrasia*, see, e.g., Horowitz (2014), Greco (2014), and Kearl (2020).

25 See Markie (2013; 2015), Wedgewood (2012), and Kearl (2022; forthcoming) for related discussions of inferential competence.

26 An anonymous referee wonders whether one *must* accept this point about the role of ability-constituting knowledge *of inference* in cases like Aki’s in order to accept the corresponding, quasi-Anscombian thesis about the role of ability-constituting knowledge of action. Strictly speaking, these two theses about action and inference are modular but mutually supportive. What I hope is that a neutral reader views my arguments as providing broad abductive support for accepting both, and so thinking that ability-constituting knowledge plays a special and structurally similar role in epistemology and the theory of action.

27 The so-called “Ramsey test”, derived from Ramsey (1929), treats one’s commitment to indicative conditionals as a kind of marker for how agents would change their attitudes in light of various suppositions. But on these sorts of views, indicative conditionals are non-truth-functional; “knowing” such conditionals, then, is a matter of encoding an agent’s propensities to accept certain propositions on the supposition that others hold. For contemporary proponents, see Edgington (1991; 2009), Sturgeon (2020).

28 For more on intellectualism, see Stanley & Williamson (2001), Stanley (2011), Bengson & Moffett (2011), Pavese (2016a; 2016b), and Waights Hickman (2019). And for more on anti-intellectualism, see Setiya (2012), Dickie (2012), Hyman (1999), and Habgood-Coote (2018). Some of these views shade into one another, but my aim is not to provide a taxonomy of them.

29 Frost (2019, Sect. 6.4) argues that knowing what one is doing as one does it shares certain features with both knowledge-how and knowledge-that without being reducible to either (Anscombian knowledge “deserves its own epistemic category” (333)). He views Anscombian knowledge and knowledge-how as a species of a common genus: “intelligent capacity” (331). Although my proposal may differ in terms of what explains what, I take it to be similar in spirit, insofar as ability-constituting knowledge is not *simply* knowledge-how, ordinarily understood. To that end, it’s worth noting some authors, more or less by *fiat*, rule out ability-constituting knowledge of *inference* as a form of knowledge-how, if only because inference is a “theoretical” concern, rather than a “practical” one. For instance, Hawley (2003), Setiya (2012), and Löwenstein (2017) accept the claim that S knows how to X only if S could X *intentionally*. I am, admittedly, not tempted to think that ability-constituting knowledge of inference leaves a residue of intention, but I’m bracketing the view that “actions” and “intentions” might be understood in a thoroughly deflationary way so as to treat certain inferences or judgments as intentional actions without collapsing into a form of doxastic voluntarism. This view may, at bottom, be no more of a terminological variant on my own suggestion, but I am trying to remain as neutral as possible on the nature of intentions per se, so I will not discuss it at length. Thanks to Juan Comesaña for raising this possibility.

30 Wither inferential knowledge of action? Sarah Paul (2009) argued that sometimes agents who act intentionally have knowledge of what they are doing as they do it, but this knowledge, when an agent has it, is merely *inferentially justified*. The thought seems to be that agents have a kind of privileged but fallible access to the contents of their own decisions, from which they can infer what they are (now) doing. Paul seems to write as though this is the *strongest epistemic position* an agent can be in with respect to what she is doing as she does it. To the extent, though, that one is sympathetic to these criticisms because of cases of absent-mindedness, cases of inarticulability, and the like, the arguments in this and the last section recommend a different perspective. Rather than rejecting a core project within the Anscombian tradition of finding necessary epistemic conditions on intentional action, my arguments suggest that the epistemic constraints on intentional action should be deflated.
